# Clinical burden of autosomal dominant polycystic kidney disease

**DOI:** 10.18632/aging.102858

**Published:** 2020-02-24

**Authors:** Peir-Haur Hung, Chien-Hung Lin, Kuan-Yu Hung, Chih-Hsin Muo, Mu-Chi Chung, Chao-Hsiang Chang, Chi-Jung Chung

**Affiliations:** 1 Department of Internal Medicine, Ditmanson Medical Foundation Chiayi Christian Hospital, Chia-Yi, Taiwan; 2 Department of Applied Life Science and Health, Chia-Nan University of Pharmacy and Science, Tainan, Taiwan; 3 Division of Pediatric Immunology and Nephrology, Department of Pediatrics, Taipei Veterans General Hospital, Taipei, Taiwan; 4 Institute of Clinical Medicine, National Yang-Ming University, Taipei, Taiwan; 5 Department of Pediatrics, Zhongxing Branch, Taipei City Hospital, Taipei, Taiwan; 6 College of Science and Engineering, Fu Jen Catholic University, New Taipei, Taiwan; 7 Department of Internal Medicine, National Taiwan University Hospital, Taipei, Taiwan; 8 Management Office for Health Data, China Medical University Hospital, Taichung, Taiwan; 9 Division of Nephrology, Department of Medicine, Taichung Veterans General Hospital, Taichung, Taiwan; 10 Department of Urology, China Medical University Hospital, Taichung, Taiwan; 11 Department of Medical Research, China Medical University Hospital, Taichung, Taiwan; 12 Department of Public Health, China Medical University, Taichung, Taiwan

**Keywords:** autosomal dominant polycystic kidney disease, hemorrhagic stroke, end-stage renal disease, all-cause mortality, time-dependent Cox proportional hazard regression

## Abstract

There are no specific therapies for autosomal dominant polycystic kidney disease (ADPKD), and clinical data evaluating the effects of non-specific therapies on ADPKD patients are scarce. We therefore evaluated those effects using data from a longitudinal health insurance database collected from 2000-2010. We individually selected patients with and without ADPKD from inpatient data files as well as from the catastrophic illness registry in Taiwan based on 1:5 frequency matching for sex, age, and index year. The hazard ratios (HR) of all-cause mortality, ischemic stroke, hemorrhagic stroke and end-stage renal disease (ESRD) in ADPKD inpatients were elevated as compared to the controls. Similarly, ADPKD patients from the catastrophic illness registry had an increased risk of hemorrhagic stroke and ESRD. Allopurinol users also had an increased risk of all-cause mortality. The HR for developing ESRD after medication exposure was 0.47-fold for statin and 1.93-fold for pentoxifylline. These results reveal that patients with ADPKD (either inpatient or from the catastrophic illness registry) are at elevated risk for hemorrhagic stroke and ESRD, and suggest that allopurinol and pentoxifylline should not be prescribed to ADPKD patients due to possible adverse effects.

## INTRODUCTION

Autosomal dominant polycystic kidney disease (ADPKD) is the most common hereditary chronic kidney disease (CKD) and can cause end-stage renal disease (ESRD), which is associated with cardiovascular morbidity and mortality [1]. ADPKD patients have a 1.6 to 3.2-fold higher risk of all-cause mortality than the general population, with cardiac-related deaths being the most common [2, 3]. There are no specific therapies for ADPKD; thus, clinical interventions focus on non-specific approaches to slow CKD progression [4–6].

ADPKD increases cardiovascular morbidity and mortality and its clinical management is more restricted than that for other forms of CKD. Inhibition of the renin-angiotensin-aldosterone system with an angiotensin converting enzyme inhibitor (ACEI) or an angiotensin II receptor blocker (ARB) delays CKD progression in patients with or without diabetes and stage 1–3 CKD, and also in patients without diabetes with stage 4 CKD [7–12]. In a small number of patients with ADPKD, statins improve renal blood flow and renal function [13]. Monotherapy with pentoxifylline decreases proteinuria excretion in patients with proteinuric diabetic and non-diabetic kidney disease [14, 15]. Previous studies reported associations between high serum uric acid levels and early-onset hypertension, large kidney volume, and increased risk of ESRD [16] or progression of renal dysfunction in ADPKD patients [17].

In our retrospective cohort study here, we used a nationwide inpatient database to investigate the clinical burden of ADPKD patients, including all-cause mortality, ischemic stroke, hemorrhagic stroke, and ESRD. We also conducted a sensitivity analysis to compare our results with the catastrophic illness registry. Finally, our study is the first to evaluate the effects of non-specific medications (ACEI, ARB, statins, pentoxifylline and allopurinol) on clinical burden risk for ADPKD patients.

## RESULTS

### Inpatient database

We analyzed data from 4342 ADPKD patients and performed 21710 comparisons. The mean age of the study population was 58.1 years (standard deviation=17.2). There were a few more men in the ADPKD group than women (55.2% vs. 44.8%) (Table 1). Compared to controls, more patients with ADPKD lived in provinces and urban villages, more lived in southern and eastern Taiwan, and more had comorbidities (all p values < 0.05). During the study period of 2000-2010, the ADPKD group had higher incidence of all-cause mortality (26.32 vs. 10.28 person-years), ischemic stroke (15.39 vs. 8.96 per 1000 person-years), hemorrhagic stroke (4.95 vs. 1.70 per 1000 person-years), and ESRD (66.21 vs. 1.18 per 1000 person-years) compared to controls (Table 2). After adjusting for age, sex, geographical area of residence and all comorbidities, the hazard ratios (HRs) of all-cause mortality, ischemic stroke, hemorrhagic stroke and ESRD in the ADPKD group were 2.47, 1.56, 3.19, and 33.1, respectively, compared to controls [corresponding to 95% confidence intervals (CIs) of 2.19–2.78, 1.35–1.81, 2.41–4.22, and 27.6–39.8, respectively].

**Table 1:** Demographic and clinical characteristics of patients with autosomal dominant polycystic kidney disease (ADPKD) and controls.

**Characteristic**	**Inpatient patients**		**P-value**		**Catastrophic illness patients**		**P-value**
	**No. (%) of individuals**				**No. (%) of individuals**		
	**With ADPKD (N=4342)**	**Control (N=21710)**			**With ADPKD (N=651)**	**Control (N=3255)**	
Gender			0.99				0.99
Female	1946 (44.8)	9730 (44.8)			336 (51.6)	1680 (51.6)	
Male	2396 (55.2)	11980 (55.2)			315 (48.4)	1575 (48.4)	
Age Group			0.99				0.99
20-39	696 (16.0)	3480 (16.0)			190 (29.2)	950 (29.2)	
40-59	1673 (38.5)	8365 (38.5)			350 (53.8)	1750 (53.8)	
≥60+	1973 (45.5)	9865 (45.5)			111 (17.1)	555 (17.1)	
Mean (SD)	58.1 (17.2)	58.0 (17.2)	0.64		47.3 (12.8)	47.2 (12.9)	0.86
Urbanization			0.0002				0.02
Provinces	1175 (27.1)	6105 (28.1)			218 (33.5)	1042 (32.0)	
Counties	1399 (32.2)	6334 (29.2)			234 (35.9)	1020 (31.3)	
Districts	716 (16.5)	3519 (16.2)			86 (13.2)	551 (16.9)	
Urban villages	1052 (24.2)	5752 (26.5)			113 (17.4)	642 (19.7)	
Geography			0.003				<0.0001
North	1936 (44.6)	9545 (44.0)			312 (47.9)	1553 (47.7)	
Central	818 (18.8)	4371 (20.1)			89 (13.7)	629 (19.3)	
South	1298 (29.9)	6621 (30.5)			181 (27.8)	916 (28.1)	
East	290 (6.68)	1173 (5.40)			69 (10.6)	157 (4.82)	
Income (NTD)			0.76				0.19
<18000	1912 (44.0)	9637 (44.4)			243 (34.3)	1339 (41.1)	
18000-34999	1933 (44.5)	9539 (43.9)			311 (47.8)	1468 (45.1)	
≥35000	497 (11.5)	2534 (11.7)			97 (14.9)	448 (13.8)	
Comorbidity							
Diabetes	489 (11.3)	1114 (5.13)	<0.0001		63 (9.69)	292 (8.97)	0.57
Hypertension	1969 (45.4)	1999 (9.21)	<0.0001		461 (70.8)	635 (19.5)	<0.0001
Hyperlipidemia	233 (5.37)	393 (1.81)	<0.0001		180 (27.7)	464 (14.3)	<0.0001
CAD	548 (12.6)	1041 (4.80)	<0.0001		87 (13.4)	258 (7.93)	<0.0001
Atrial fibrillation	119 (2.74)	184 (0.85)	<0.0001		6 (0.92)	15 (0.46)	0.14
Congestive heart failure	260 (5.99)	330 (1.52)	<0.0001		22 (3.38)	36 (1.11)	<0.0001
Obesity	5 (0.12)	4 (0.02)	0.009		0 (0.00)	4 (0.12)	0.99
Gouty arthritis	247 (5.69)	235 (1.08)	<0.0001		74 (11.4)	107 (3.29)	<0.0001
Medications							
ACE-Is					207 (31.8)	331 (10.2)	<0.0001
ARBs					392 (60.2)	405 (12.4)	<0.0001
Statins					159 (24.4)	393 (12.1)	<0.0001
Allopurinol					71 (10.9)	48 (1.47)	<0.0001
Pentoxifylline					113 (17.4)	92 (2.83)	<0.0001

Chi-square test and *t*-test; CAD, coronary artery disease; SD, standard deviation; Medicine users were defined during the study period

**Table 2:** Risk of various outcomes in ADPKD patients compared to controls.

**Variable**	**Inpatient database^1^**		**Catastrophic illness patients^2^**	
	**With ADPKD**	**Control**	**With ADPKD**	**Control**
N	4310	21550	651	3255
Person-years	16373	121626	2540	14774
All-cause mortality				
Event no	431	1250	15	53
Incidence density	26.32	10.28	5.90	3.59
Crude HR (95% CI)	2.54 (2.27-2.83)***	Ref.	1.78 (0.99-3.17)	Ref.
Adjusted HR (95% CI)	2.47 (2.19-2.78)***	Ref.	1.71 (0.84-3.48)	Ref.
Ischemic stroke				
Event no	252	1090	7	49
Incidence density	15.39	8.96	2.76	3.32
Crude HR (95% CI)	1.69 (1.47-1.94)***	Ref.	0.85 (0.38-1.87)	Ref.
Adjusted HR (95% CI)	1.56 (1.35-1.81)***	Ref.	0.49 (0.20-1.22)	Ref.
Hemorrhagic stroke				
Event no	81	207	14	8
Incidence density	4.95	1.70	5.51	0.54
Crude HR (95% CI)	2.87 (2.22-3.72)***	Ref.	9.83 (4.12-23.5)***	Ref.
Adjusted HR (95% CI)	3.19 (2.41-4.22)***	Ref.	4.41 (1.41-13.8)*	Ref.
ESRD				
Event no	1084	144	116	5
Incidence density	66.21	1.18	45.66	0.34
Crude HR (95% CI)	49.1 (41.3-58.5)***	Ref.	132 (54.0-324)***	Ref.
Adjusted HR (95% CI)	33.1 (27.6-39.8)***	Ref.	56.4 (21.4-146)***	Ref.

^1^Adjusted for age, gender, geography, and all comorbidity using extended Cox proportional hazard regression.

^2^Adjusted for age, gender, geography, all comorbidity, and medications using Cox proportional hazard regression.

* p<0.05, *** p<0.001.

### Longitudinal health insurance database for catastrophic illness patients (LHID-CIP)

In total, data from 651 patients with ADPKD and 3255 age- and sex-matched controls were analyzed. Among the ADPKD group, there were female patients more than males (51.6% vs. 48.4%, respectively), and the mean age was 47.3 years (standard deviation=12.8) (Table 1).

Compared to controls, more than 75% of patients with ADPKD lived in northern and southern Taiwan, had more comorbidities, including hypertension, hyperlipidemia, coronary artery disease (CAD), congestive heart failure, and gouty arthritis. In addition, ADPKD patients were prescribed various medications such as ACEIs, ARBs, statins, allopurinol, and pentoxifylline.

During the study period, the ADPKD group had a 1.78-fold higher incidence of all-cause mortality (5.90 vs. 3.59 person-years), 0.85-fold higher incidence of ischemic stroke (2.76 vs. 3.32 per 1000 person-years), 9.83-fold higher incidence of hemorrhagic stroke (5.51 vs. 0.54 per 1000 person-years), and 132-fold higher incidence of ESRD (45.66 vs. 0.34 per 1000 person-years) compared to the controls (Table 2). After adjusting for age, sex, geographical area of residence, all comorbidities, and all medications, the ADPKD group had increased risk of hemorrhagic stroke and ESRD (HR=4.41 and 56.4, 95% CI, 1.41−13.8 and 21.4−146, respectively).

Table 3 shows the effects of medications on each outcome in the ADPKD group. ADPKD patients receiving allopurinol treatment had a 6.44-fold higher risk of all-cause mortality than no-treatment controls (95% CI, 1.72−24.2). In addition, ADPKD patients receiving pentoxifylline treatment had an increased risk of ESRD (HR = 1.93, 95% CI, 1.20−3.09), whereas those who received statin treatment had a lower risk of ESRD (HR = 0.47, 95% CI, 0.27−0.85). There were no medication effects on ischemic or hemorrhagic stroke.

**Table 3:** Association between clinical medications and ADPKD patient outcomes using time-dependent Cox proportional hazard regression.

**Medication (yes vs. no)**	**All-cause mortality**		**Ischemic stroke**		**Hemorrhagic stroke**		**ESRD**	
	**Crude**	**Adjusted**	**Crude**	**Adjusted**	**Crude**	**Adjusted**	**Crude**	**Adjusted**
ACE-Is	3.44 (1.19-9.96)*	2.35 (0.69-8.07)	14.5 (1.65-126)*	9.50 (0.92-97.9)	2.26 (0.76-6.73)	1.88 (0.57-6.18)	1.49 (1.01-2.20)*	1.29 (0.86-1.94)
ARBs	0.78 (0.28-2.18)	0.70 (0.20-2.50)	0.66 (0.14-3.04)	0.48 (0.08-2.74)	1.23 (0.42-3.63)	1.05 (0.32-3.52)	1.72 (1.18-2.53)**	1.26 (0.84-1.90)
Statins	1.08 (0.30-3.87)	0.76 (0.18-3.32)	1.64 (0.30-8.91)	1.93 (0.26-14.2)	1.72 (0.52-5.66)	1.04 (0.26-4.25)	0.73 (0.43-1.24)	0.47 (0.27-0.85)*
Allopurinol	10.2 (3.55-29.3)***	6.44 (1.72-24.2)**	NA	NA	2.42 (0.53-11.0)	1.45 (0.26-8.09)	1.72 (0.92-3.21)	1.12 (0.56-2.24)
Pentoxifylline	3.41 (1.15-10.1)*	2.97 (0.75-11.8)	1.03 (0.12-8.92)	2.00 (0.17-23.6)	3.86 (1.25-11.9)*	3.27 (0.94-11.4)	2.33 (1.51-3.61)***	1.93 (1.20-3.09)**

Adjusted for age, gender, geography, all comorbidity, and medications.

* p<0.05, ** p <0.01, *** p<0.001

**Figure 1:**
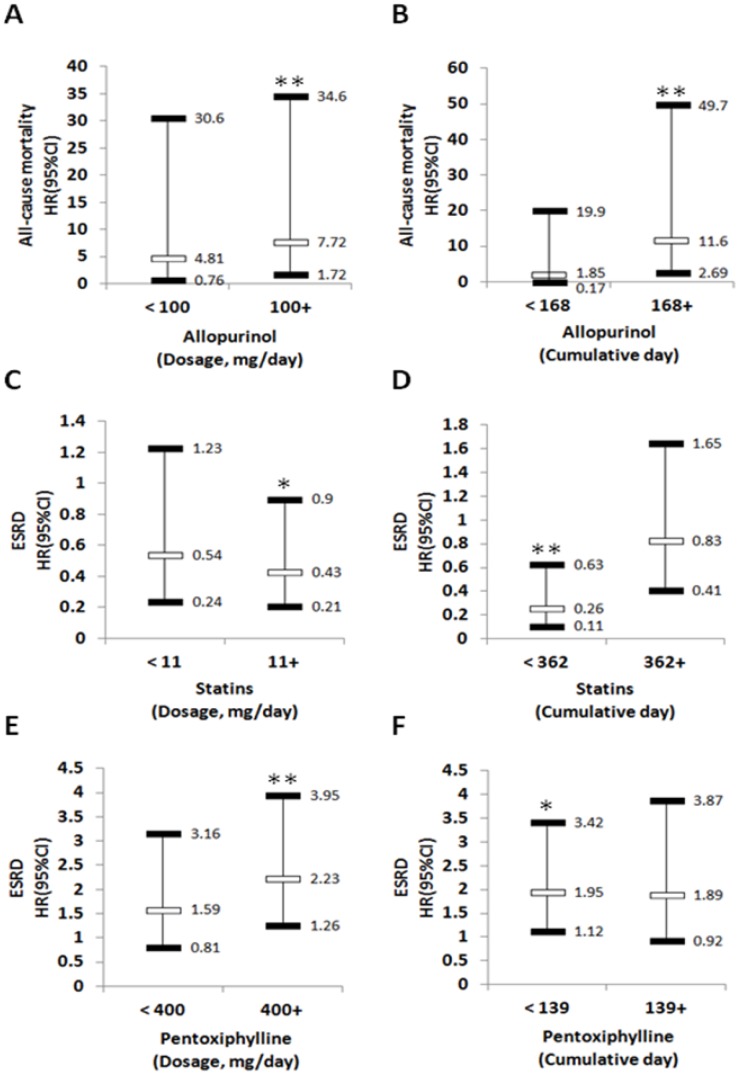
**The association of medication dose and duration with various outcomes.** Risk of all-cause mortality according to dosage (**A**) or cumulative (**B**) of Allopurinol use. Risk of ESRD according to dosage (**C**) or cumulative (**D**) of Statins use. Risk of ESRD according to dosage (**E**) or cumulative (**F**) of Pentoxiphylline use. Adjusted for age, gender, geography, all comorbidity and medications. ESRD: end-stage renal disease; HR: hazard ratio; CI: Confidence interval. * p<0.05, ** p <0.01.

Multivariable-adjusted time-dependent Cox proportional hazard regression showed that allopurinol doses ≥100 mg/day were associated with 7.72 (95% CI, 1.72−34.6) hazards of all-cause mortality compared with allopurinol non-users (Figure 1A). Longer allopurinol use duration was associated with higher hazard of all-cause mortality (HR = 11.6, 95% CI, 2.69–49.7; P<0.01) compared with allopurinol non-users (Figure 1B). Compared with statin non-users, higher statin doses of ≥ 11 mg/day (HR = 0.43, 95% CI, 0.21–0.9) or shorter statin use duration (HR = 0.26, 95% CI, 0.11–0.63) were associated with lower hazard of incident ESRD (Figure 1C and 1D). In this analysis, we also found that, compared with pentoxifylline non-users, higher doses of pentoxifylline ≥400 mg/day (HR = 2.23, 95% CI, 1.26–3.95) or shorter use duration of pentoxifylline (HR = 1.95, 95% CI, 1.12–3.42) were associated with decreased risk of ESRD (Figure 1E and 1F).

## DISCUSSION

To the best of our knowledge, the present study is the first nationwide population-based cohort study to compare the incidence rates of all-cause mortality, ischemic stroke, hemorrhagic stroke, and ESRD in patients with ADPKD with matched controls from the general population. More specifically, we used multivariate regression analysis to retrospectively compare the development of the above outcomes between patients with ADPKD and controls without ADPKD. Our results indicated that both inpatients and patients with ADPKD had increased risks of hemorrhagic stroke and ESRD. In addition, the use of statins correlated with a reduced risk of ESRD. However, the use of allopurinol and pentoxifylline were associated with an increased risk of all-cause mortality and ESRD, respectively.

In our analysis, the incidence of hemorrhagic stroke was higher in the patients with ADPKD compared to controls, with an adjusted HR of 3.19 for the inpatients and 4.41 for those with a catastrophic illness. An underlying defect in the connective tissue matrix has been associated with intracranial aneurysms, hepatic cysts, diverticulosis, spontaneous coronary artery dissection, and other cardiovascular abnormalities in patients with ADPKD [18–20]. The prevalence of intracranial aneurysm in patients with ADPKD is also higher than that among the general population (9–12% vs. 2–3%), and aneurysm rupture causing subarachnoid or intracerebral hemorrhage is among the most serious complications of ADPKD [18–20]. However, little is known about the incidence of hemorrhagic stroke in patients with ADPKD compared to those without ADPKD. Thus, further studies on therapies to prevent aneurysm rupture and reduce the risk of hemorrhagic stroke may improve outcomes during the transition from CKD to ESRD in ADPKD patients.

ADPKD patients had an increased incidence of ESRD in our study. ADPKD differs from most other causes of ESRD because it can be detected early in life, and approximately 50% of affected individuals experience advanced kidney failure by 60 years of age [21]. Thus, a multidrug approach might benefit ADPKD patients, not only to slow CKD progression, but also to decrease cardiovascular complications, the major cause of morbidity and mortality in these patients [22]. Aggressive blood pressure control with an ACEI or ARB forms the basis of this therapy. Since there is currently no clinically approved specific therapy for ADPKD [23], current consensus guidelines [24] recommend managing hypertension, decline in renal function and renal complications to prevent the progression of ADPKD to ESRD. Our analysis concentrated on cardiorenal protection as a whole and found that the use of statins might moderately reduce the incidence of ESRD in patients with ADPKD without increasing the risk of death. Improvements in endothelial dysfunction in the setting of simvastatin treatment for ADPKD were demonstrated in a prospective cohort study of 16 patients [25], although no direct clinical endpoint was apparent. A pediatric study of 110 participants treated with lisinopril and randomized to receive a placebo or pravastatin for three years demonstrated benefits in height-corrected total kidney volume expansion associated with randomization to pravastatin [26]. In contrast, a small randomized open-label study of 49 patients with ADPKD who received pravastatin therapy for two years showed no effect on renal dysfunction [27]. Further research is needed to investigate the mechanisms underlying the effects of statins on ADPKD.

A 6.44-fold increased risk of mortality was associated with the use of allopurinol in our ADPKD cohort with gouty arthritis. In patients with ADPKD and CKD, serum uric acid may be an independent factor for renal progression [28], and it has been associated with the earlier onset of larger kidney volume, hypertension, and increased risk of ESRD [29–31]. Recent epidemiologic studies have shown a relationship between uric acid level and progression of kidney disease [16, 32]; however, to the best of our knowledge, no data have been published characterizing the effect of allopurinol on the long-term progression of CKD in patients with ADPKD. While several previous studies evaluated the influence of allopurinol treatment on all-cause mortality, they reported conflicting results [33–35]. Yang et al. found that CKD was strongly associated with allopurinol hypersensitivity (OR = 1.49, 95% CI, 1.38−1.61; P < 0.001) and all-cause mortality (OR = 2.20, 95% CI, 1.69−2.87; P < 0.001) in Taiwan [36]. Additionally, older age, CKD and cardiovascular disease are non-genetic risk factors for allopurinol hypersensitivity and hypersensitivity-related mortality [37, 38]. Our findings here showed a consistent effect for allopurinol use on all-cause mortality in ADPKD patients. However, studies with a longer observation period are needed to confirm our findings.

Pentoxifylline is used to treat peripheral vascular disease and microcirculatory disorders. In addition to its hemorrheologic activity, pentoxifylline provides antiproliferative and anti-inflammatory effects that are organoprotective, reduces proteinuria in patients with diabetic and non-diabetic proteinuric kidney disease [39, 40], and improves renal function [41, 42]. However, our analysis here showed that pentoxifylline increased the risk of ESRD for ADPKD patients.

There are several limitations to the present study. First, ADPKD patients applying for catastrophic illness certificates were selected on the basis of their first claim date; therefore, there may be a time delay. However, as the NHIRD is a deidentified secondary database, it is difficult to determine whether or not underestimation occurred. Second, data on personal habits such as lifestyle, smoking, alcohol use, body weight, and disease severity are not available in the NHIRD. These factors are also important and could influence both propensity for active gout and other outcomes. Third, we did not have imaging data for ADPKD patients, which might have been potential confounders. Fourth, a prospective randomized control clinical trial would be the gold standard to compare outcomes. Thus, this study did not demonstrate the causal link (but only concomitance) between allopurinol use and mortality. The same applies to pentoxifylline use and the risk of ESRD. Fifth, the causal link between allopurinol and mortality as well between pentoxifylline and ESRD cannot be demonstrated in this study. Finally, diagnosis bias and loss to follow-up are inherent in cohort studies [43]. In this study, the cohorts were based on the Registry for Catastrophic Illness Patient Database and in-hospital patients, with well-established diagnoses for ADPKD and ESRD [44], which helps avoid diagnostic bias.

In conclusion, to the best of our knowledge, this is the first report of an association between ADPKD and the risk of hemorrhagic stroke and ESRD with matched controls from the general population. CKD increases mortality, and CKD is accompanied by hyperuricaemia which is treated by allopurinol; similarly, it is proteinuria that increases the risk of ESRD, but not the treatment of proteinuria with pentoxifylline. The risk of developing ESRD after medication exposure was reduced for statins and increased for pentoxifylline. In addition, the use of allopurinol was associated with an increased risk of all-cause mortality in ADPKD patients. Health care professionals should be aware of this risk when treating ADPKD patients.

## MATERIALS AND METHODS

### Data source

We used the inpatient database and longitudinal health insurance database for catastrophic illness patients from the National Health Insurance Research Database (NHIRD) in Taiwan. The NHIRD was established by the Taiwan National Health Insurance Administration Ministry of Health and Welfare based on the National Health Insurance program in Taiwan, and it is maintained by the Taiwan National Health Research Institutes. The NHI program covers over 99% of the population in Taiwan. The inpatient database included all hospital claims from 1996 to 2011, and the LHID-CIP included all inpatient claims, outpatient claims, and medical treatment of patients with catastrophic illnesses from 1997 to 2011. Twenty-nine diseases are classified as being catastrophic illnesses in Taiwan, including ESRD, ADPKD, and organ transplant. Patients are diagnosed as having a catastrophic illness according to guidelines from the Bureau of NHI. These patients can then apply for a catastrophic illness card, which has to be formally reviewed by specialist physicians, and are then exempt from copayments. In the NHIRD, all diseases are defined according to the International Classification of Diseases, Ninth Revision, Clinical Modification (ICD-9-CM). Medications are defined based on the Anatomical Therapeutic Chemical (ATC) classification system, which was established by the World Health Organization Collaborating Centre for Drug Statistics Methodology (http://www.whocc.no). All patient data are deidentified and encrypted before being released for research purposes. This retrospective cohort study was approved by the Research Ethics Committee of China Medical University Hospital (IRB CMUH104-REC2-115(CR-4)).

### Study subjects

### Inpatient database

Data for patients with admission for ADPKD (ICD-9-CM 753.12, and 753.13) from 2000-2010 were collected as the ADPKD cohort (n = 6019). The date of this admission was defined as the index date. The exclusion criteria were: 1) age < 20 years (n = 222); 2) a history of stroke (n = 932); 3) ESRD (n = 515); and 4) kidney transplant (n = 8). Dara from patients without ADPKD or any of the exclusion criteria were used as controls, and were randomly assigned with an index date as per the ADPKD cohort. Five controls were randomly selected for each patient and frequency matched by age group (20-39, 40-59 and ≥60 years), sex, and index year.

### LHID-CIP

**Figure 2:**
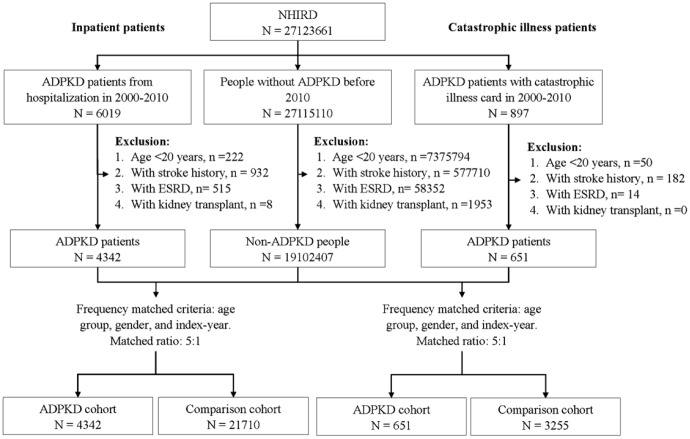
**Flow chart of study recruitment.**

Data for 897 patients with catastrophic illness cards for ADPKD from 2000-2010 were used as the ADPKD cohort. The date of application for a catastrophic illness card was defined as the index date. The exclusion criteria were: 1) age < 20 years (n = 50); 2) a history of stroke (n = 182); 3) ESRD (n = 14); and 4) kidney transplant (n = 0). Data from patients without a catastrophic illness card for ADPKD or any of the exclusion criteria were used as controls. They were randomly assigned with an index date as the ADPKD cohort. Five controls were randomly selected for each patient and frequency matched by age group (20-39, 40-59 and ≥60 years), sex, and index-year (Figure 2).

### Outcome and covariate

All of the subjects were followed from the index date until a study outcome of death, stroke [including ischemic stroke (ICD-9-CM 433-438), and hemorrhagic stroke (ICD-9-CM 430-432)], or the development of ESRD (ICD-9-CM 585), whichever occurred first. To improve accuracy, outcome diagnoses were defined from catastrophic illness files. The detailed recruitment procedure is shown in Figure 2. Covariates included demographics, baseline comorbidities, and medications, and demographic data included sex, age, urbanization, geographical area of residence, and income. Comorbidities (ICD-9-CM) included diabetes (250), hypertension (401-405), hyperlipidemia (272), coronary artery disease (CAD: 410-414), atrial fibrillation (427.31), congestive heart failure (428), obesity (278.0), and gouty arthritis (274). Medications (ATC) included ACEIs (C09AA, C09BA, and C09BB), ARBs (C09CA, C09DA, C09DB, and C09DX), statins (C10AA), allopurinol (M04AA01), and pentoxifylline (C04AD03). Patients who were prescribed medications during the study period were defined as users.

### Statistical analysis

SAS software (version 9.4 for Windows; SAS Institute Inc., Cary, NC, USA) was used for all analyses. A two-tailed P value of < 0.05 was considered to be statistically significant. Differences in categorical variables between patients with and without ADPKD were tested using the chi-square test, and continuous variables were compared using the *t*-test. All-cause mortality, and the incidence rates of ischemic stroke, hemorrhagic stroke, and ESRD were calculated in the ADPKD and comparison cohorts. HR and 95% CIs of different outcomes were evaluated using a Cox proportional hazard model. The multivariate model was adjusted for other potential covariates. The assumption of the Cox proportional hazard model was certified through testing the scaled Schoenfeld residuals. The results of testing in the LHID-CIP did not violate the assumption of each outcome. However, in the inpatient database, there was an association between Schoenfeld residuals for ADPKD and follow-up time for each outcome. Therefore, we used an extended Cox regression model to estimate the risk of different outcomes between the patients with ADPKD in the inpatient database. The effect of medications was assessed only in patients with ADPKD using time-dependent Cox proportional hazard regression analysis.
